# Rapid identification of major *Mycobacterium* species by loop-mediated isothermal amplification assay using novel species-specific genomic targets

**DOI:** 10.3389/fcimb.2025.1653602

**Published:** 2025-09-17

**Authors:** Yuanwu Zou, Zhuo Wang, Zihan Wei, Guanghong Bai, Xiaolin Wang, Shaoyi Qu, Guowei Zhong, Yanbin Gao

**Affiliations:** ^1^ Department of Clinical Laboratory, Shaanxi Provincial Hospital of Tuberculosis Prevention and Treatment, The Fifth People’s Hospital of Shaanxi Province, Xi’an, China; ^2^ Department of Epidemiology and Biostatistics, School of Public Health, Xi’an Jiaotong University Health Science Center, Xi’an, China; ^3^ Department of Clinical Laboratory, Shaanxi Provincial People’s Hospital, Xi’an, China; ^4^ Tuberculosis Medicine Department II, Shaanxi Provincial Hospital of Tuberculosis Prevention and Treatment, The Fifth People’s Hospital of Shaanxi Province, Xi’an, China; ^5^ Shaanxi Provincial Hospital of Tuberculosis Prevention and Treatment, The Fifth People’s Hospital of Shaanxi Province, Xi’an, China

**Keywords:** nontuberculous mycobacteria, mycobacterium tuberculosis, loop-mediated isothermal amplification, species identification, clinical validation

## Abstract

**Background:**

Rapid and precise identification of *Mycobacterium* species is critical for appropriate clinical management and epidemiological surveillance. However, conventional methods often fail to differentiate closely related nontuberculous mycobacteria (NTM) species, limiting their clinical utility.

**Methods:**

We developed a multiplex loop-mediated isothermal amplification (LAMP) assay targeting newly identified species-specific genomic markers for simultaneous detection of *Mycobacterium tuberculosis* complex (MTBC) and six clinically important NTM species. Analytical performance was assessed using serial dilutions of bacterial cultures and 36 reference strains. Clinical validation was performed on 52 cultured isolates and 349 sputum samples, compared to GeneXpert MTB/RIF and a commercial PCR-reverse dot blot assay.

**Results:**

The assay showed high analytical sensitivity, with limits of detection ranging from 76.013 CFU/mL (95% CI: 60.329-113.924 CFU/mL) for MTBC to 166.602-690.629 CFU/mL for NTM species. All reference strains were correctly identified with no cross-reactivity. Among the clinical isolates, all targeted species were accurately detected. One isolate misidentified as *M. abscessus* by an ITS-based assay was confirmed by sequencing to be *M. massiliense*, demonstrating the assay’s superior discriminatory capacity. For sputum samples, the assay achieved 90.32% sensitivity and 97.55% specificity for MTBC, with an overall agreement of 93.70% (κ = 0.8740).

**Conclusion:**

This multiplex LAMP assay offers a rapid, accurate, and field-deployable tool for species-level identification of MTBC and major NTM pathogens. Its simplicity, stability, and compatibility with low-resource settings support its application in routine diagnostics and decentralized tuberculosis programs.

## 1 Introduction

The genus *Mycobacterium* comprises diverse species, including the *Mycobacterium tuberculosis* complex (MTBC) and nontuberculous mycobacteria (NTM), which pose significant threats to global public health. Tuberculosis (TB), caused by MTBC, remains a leading cause of mortality worldwide, with an estimated 10.8 million new cases and 1.25 million deaths reported in 2023 by World Health Organization (W.H.O, 2024). Concurrently, NTM infections have emerged as clinically significant pathogens, particularly among immunocompromised individuals, complicating differential diagnosis due to overlapping clinical manifestation with TB (Prevots and Marras, 2015; Ratnatunga et al., 2020). The accurate identification of MTBC and NTM is of critical importance in both clinical and public health contexts. MTB and NTM infections often present with similar clinical symptoms, yet their treatment regimens differ significantly (Tortoli, 2009). NTM species are typically resistant to first-line anti-tuberculosis drugs, necessitating species-specific therapy guided by identification results. For example, *Mycobacterium avium complex* requires macrolide-based regimens, whereas *Mycobacterium kansasii* responds to rifampicin-containing protocols (Jhun et al., 2018; Moon et al., 2019). Misdiagnosis may lead to iatrogenic harm from inappropriate TB chemotherapy or delayed NTM-targeted treatment (Skendros et al., 2014), underscoring the direct impact of diagnostic accuracy on patient outcomes and healthcare costs.

Molecular techniques like PCR and sequencing represent significant advancements over culture-based methods, offering improved speed and accuracy for mycobacterial identification (Gupta et al., 2010; Wang et al., 2015; Chae et al., 2017). However, their reliance on sophisticated infrastructure, skilled personnel, and high operational costs limits accessibility in resource-constrained settings, where TB and NTM burdens are often highest. Current PCR-based NTM identification strategies predominantly target the 16S-23S ribosomal internal transcribed spacer (ITS), a region selected for its interspecies variability (Ngan et al., 2011; Pourahmad et al., 2019). Nevertheless, ITS exhibits insufficient discriminatory power for closely related species like the *M. abscessus complex*, and requires downstream sequencing for confirmation, thereby perpetuating infrastructure dependency. Additionally, ITS-based assays cannot resolve polyclonal infections without supplementary gene targets, further increasing complexity and turnaround time (Almeida and Araujo, 2013). These limitations collectively highlight the unmet need for target-specific, rapid multiplex assays capable of decentralized species differentiation.

To address this diagnostic gap, we developed a multiplex loop-mediated isothermal amplification (LAMP) assay targeting genomic markers beyond *ITS*, enabling simultaneous identification of MTBC and major NTM species within 60 minutes. We optimized species-specific primer sets to enhance discriminatory power, ensuring robust detection even in low-biomass samples. By integrating clinical validation with analytical sensitivity and specificity testing, this work advances a cost-effective, field-deployable tool capable of informing timely therapeutic decisions and improving epidemiological surveillance. The following sections detail the assay design, experimental validation, and comparative performance against gold-standard methods, underscoring its potential to transform mycobacterial diagnostics in diverse healthcare settings.

## 2 Materials and methods

### 2.1 Target selection and primer design

To enable specific identification of MTBC and the most clinically relevant NTM species, we performed a systematic bioinformatic analysis to screen for novel species-specific genomic targets. Complete genome sequences of representative strains were retrieved from the NCBI GenBank database, including at least five strains per species for MTBC, and major NTM species such as *M. avium*, *M. intracellulare*, *M. kansasii*, *M. abscessus*, *M. gordonae* and *M. fortuitum*. Comparative genome alignment was conducted using the progressive mauve algorithm and BLAST to identify conserved and species-unique regions with minimal homology to other mycobacterial or non-mycobacterial species. Candidate gene regions were further evaluated based on the following criteria: 1) presence in all sequenced strains of the target species but absent or significantly divergent in non-target species. 2) low intraspecies variability to ensure primer binding site conservation. 3) suitable length (200~500 bp) and GC content compatible with LAMP reaction conditions. 4) biological relevance or stable genomic presence to minimize potential gene loss or horizontal transfer.

Species-specific LAMP primers, including forward inner primer (FIP), backward inner primer (BIP), outer primers (F3 and B3), and loop primers (LF and LB), were designed using PrimerExplorer V5 software (Eiken Chemical Co., Ltd.) based on the conserved regions of selected targets. All primers were synthesized by a commercial provider (Sangon Biotech, Shanghai, China).

### 2.2 Preparing the target DNA

Genomic DNA was extracted from reference strains using the HiPure Bacterial DNA Kit (Magen Biotech Co., Ltd., China) according to the manufacturer’s instructions. The concentration and purity of the extracted DNA were measured using a Qubit fluorometer (Thermo Fisher Scientific, USA). Subsequently, DNA fragments for each pathogen were amplified from the genomic DNA of reference strains via PCR. The PCR products were purified and cloned into plasmid vectors (pUC-57), which were subsequently transformed into competent *Escherichia coli* cells and propagated through bacterial culture.

### 2.3 LAMP assay

Unlike the conventional liquid-phase approach, the LAMP assay employed in this study utilizes a vitrification technique to immobilize primers, enzymes, and dNTPs at the bottom of the reaction tube (Huang et al., 2024). This strategy enables the reagents to remain stable during storage and transportation at 2~8°C, thereby improving their applicability in both clinical and field settings. To simultaneously detect eight targets, the mixtures (primers, enzymes, and dNTPs) corresponding to each target were vitrified and separately preloaded into the wells of a 96-well plate. LAMP reaction was performed in a 25-μL reaction mixture containing 1×Isothermal Amplification Buffer (Nanjing Vazyme, China), 6 mM MgSO_4_, 1.4 mM dNTP, 0.2 μM each F3 and B3 primers, 1.6 μM each FIP and BIP primers, 0.8 μM each LF and LB primer, 8U *Bst* II Pro DNA Polymerase Large Fragment (Nanjing Vazyme, China), 0.5μL SYTO-9 (Thermol Fisher, USA), 5μL target DNA, and nuclease-free water. The reaction was performed SLAN-96S real-time PCR system (Hongshi Tech Co., ltd, China) under the following conditions: 63°C for 30s, with 40 cycles of 63°C for 1 min. Fluorescence signals were measured at the end of each cycle. The pan-*Mycobacterium* 16S rRNA gene was included as an internal amplification control in all reactions to exclude false negatives caused by amplification failure. In addition, genomic DNA of *M. tuberculosis* was used as a positive control in each run to monitor amplification performance.

### 2.4 Analytical sensitivity and specificity

The limit of detection (LOD) was evaluated by qualified CFU. For the LOD of qualified CFU, serial dilutions with the indicated mycobacterial numbers were spiked into the clinical non-infected sputum samples, ranging from 10 CFU/mL to 10^5^ CFU/mL (Luukinen et al., 2024). Each dilution was prepared and tested in twenty replicates to ensure statistic reliability, and the LOD was determined as the lowest dilution which yield the detection of the targets ≥95% probability.

To evaluate the inclusivity and specificity of the developed assay, Genomic DNA was extracted from other clinical respiratory pathogens, including 28 mycobacterial strains and 8 non-mycobacterial species, were used to evaluate the inclusivity and specificity of the developed assay (**Supplementary Table S1**).

### 2.5 Application of LAMP of clinical mycobacterial cultures or sputum

Due to the relatively low incidence of NTM in primary clinical specimens within our region, we employed stored culture isolates to evaluate the LAMP assay’s sensitivity and specificity for NTM detection. Specifically, MGIT and solid medium based-mycobacterial cultures, preserved from routine diagnostic workups, provided sufficient bacterial load and species diversity for performance assessment. Conversely, sputum samples, collected according to standard protocols for pulmonary tuberculosis diagnosis, were used to assess LAMP performance for the direct detection of MTBC, thereby reflecting routine clinical practice and maximizing the assay’s translational relevance.

For sputum sample processing, 2 mL of N-acetyl-L-cysteine (NALC)-2% NaOH solution was added to 1 mL of sputum in a sterile specimen cup. The mixture was vortexed thoroughly and incubated at room temperature for 10 minutes. Following liquefaction, 2 mL of the processed sample was centrifuged and washed once with sterile normal saline. The supernatant was discarded, and the pellet was resuspended in 50 μL of a heat lysis buffer (Deaou Bioscience Co., Ltd., China). The suspension was then incubated at 95°C for 10 minutes. The resulting lysate was used as the nucleic acid template for LAMP assay.

### 2.6 *Mycobacterial* species identification by commercial PCR kit

To evaluate the accuracy of the developed assay, NTM species identification was conducted in parallel using a commercially available PCR-reverse dot blot (PCR-RDB) assay kit (Yaneng Bioscience Co., Ltd., China), which has been approved by the National Medical Products Administration (NMPA) of China (Li et al., 2025). Genomic DNA was extracted from cultured isolates according to the manufacturer’s protocol, 4 µL of the extracted DNA was used for PCR amplification. Subsequently, 20 μL PCR products were used for DNA detection via a reverse dot blot platform. On the other hand, identification of MTBC was performed using the GeneXpert MTB/RIF assay (Cepheid, USA), sample processing and analysis were conducted following the standard operating protocol provided by the manufacturer.

### 2.7 Statistical analysis

Data were analyzed statistically using IVD Statistics and SPSS 28.0. Sensitivity, specificity, and overall accuracy, together with their two-sided 95% confidence intervals (CIs) based on the Wilson score method, were calculated by comparing the novel assay with the GeneXpert MTB/RIF and a commercial PCR-reverse dot blot kit (Yaneng Bioscience Co., Ltd., China). The limit of detection (LOD) was estimated using a probit regression model, where detection outcomes (positive/negative) were regressed against log10-transformed bacterial concentrations, and defined as the concentration corresponding to a 95% probability of detection.

## 3 Results

### 3.1 Target selection and LAMP primer selection

Based on the results of the bioinformatic screening, specific target sequences were amplified by conventional PCR using primers designed for each candidate region. The selected gene targets included *IS6110* for MTBC, *gyrB* for *M. avium*, *hypothetical protein XDL66245.1* for *M. intracellulare*, *hypothetical protein B1T47_13295* for *M. kansasii*, *erm (41)* for *M. abscessus*, *ITS* for *M. gordonae*, and *dnaA* for *M. fortuitum* (Liu et al., 2019). Additionally, the pan-Mycobacterium 16S rRNA gene, which is conserved across all mycobacterial species, was also included to help identify potential false-negative results due to the absence of amplification in NTM, and the detailed sequences were presented in **Supplementary Table S2**. PCR products were purified and subjected to Sanger sequencing to confirm sequence accuracy. The obtained sequences were aligned with corresponding reference genomes using BLAST to verify their identity and specificity. Only targets showing 100% identity with reference sequences and no significant homology to non-target mycobacterial or other bacterial species were selected for subsequent LAMP primer design.

After confirming that the selected target regions met the required criteria, at least seven sets of LAMP primers were designed and evaluated for each target. The final primer sets for each target were determined based on multiple validation parameters (**Table 1**), including consistent results in negative control replicates, amplification efficiency across reference strains, and performance in detecting negative clinical samples.

**Table 1:** The set of primers used for the *Mycobacterium tuberculosis* complex (MTBC) and nontuberculous mycobacteria (NTM) detection LAMP assay in this study.

Species (target)	Primers	Sequence (5’ to 3’)
MTBC (*IS6110*)	F3	GGTCGGAAGCTCCTATGA
	B3	GTTGAGCGTAGTAGGCAG
	FIP	GGGCTTGCCGGGTTTGATCAATGCACTAGCCGAGAC
	BIP	GTCCATCGAGGATGTCGAGTTGCCGCAGTACTGGTAGAGG
	LF	CAGCTCGGTCTTGTATAGGC
	LB	TGGGTCGACTGGTTCAAC
NTM (*16SrRNA*)	F3	CCGCGGTAATACGTAGGGT
	B3	ACCGGTGTTCCTCCTGAT
	FIP	CGCTCACAGTTAAGCCGTGAGATCCGGAATTACTGGGCGT
	BIP	GGCGATACGGGCAGACTAGAGTCGCATTCCACCGCTACAC
	LF	ACAAACCACCTACGAGCTCTT
	LB	ACTGCAGGGGAGACTGGA
*M. intracellulare* (*MINTM006_47990*)	F3	TCTTCCGTGGTACGAGAG
	B3	TGATACGGAGAAGGGATCG
	FIP	TGCACATCCTGCGCCAATCCATACATGGCGACCTTG
	BIP	GTAATCCGGCGTGGCTGTACTCCCACCAATAAGGCA
	LF	ATGGCGATCGCGATTAGG
	LB	CCATTCTGCTGGCCATCTC
*M. avium* (*gyrB*)	F3	GCCTGACCATCAACCTCACC
	B3	TGGTGGATGGGGTTTTTGG
	FIP	CTTCTCCTGCGCCGACTTGGCGGGTGACCAACGAAGAG
	BIP	AATCGGCTGCGCCGCATAAGTTTGACGAAGTCGACCAGG
	LF	TGTCGCTGACCACCTCG
	LB	TCAAGCACCGCACCTTC
*M. abscessus* (*erm41*)	F3	TGCCAGGGTGCTAGCCGTCGA
	B3	TGGCGAACAGGTGCTCGCT
	FIP	GAAATGGCCGTCGCGGTTCGTTCACGGTTTGCCGAG
	BIP	TGGTGGCGAGCCCACCAGGTCGGCAGCCA
	LF	GCGAGCAGGTCCGCTTCCGCTAT
	LB	ACCAAGTCACCAGCGCA
*M. kansasii*(*hypothetical protein B1T47_13295*)	F3	CCCGGTCCACTATTACGACA
	B3	CAGTGTCAGTGCAAGATCGA
	FIP	GGAACCGTTGATCGGTTGCCGTTCAGAATCGACCGCATGG
	BIP	CGCCAACATGGCAATCCGGACGGTCTTCCAGATCCAGCT
	LF	TGCGATGCAGGTTGCGG
	LB	GGCAGGCGGTACGTCAT
*M. gordonae* *(ITS)*	F3	CCCTTAGACACTTACAAACACT
	B3	TAAGGAGCACCACGAAGA
	FIP	CCTCCATCTTGGTGGTGGGCGAACGACAACGCATACA
	BIP	GTGTTGCCTCAGGACCCAATTCTGTAGTGGACGAAGACC
	LF	AGTGGTTGCGAGCATCAA
	LB	GCTTTGCCTGTTGTCGTG
*M. fortuitum* *(dnaA)*	F3	GATCGTGATTCCAACAGTGA
	B3	AGTCCTCGGATTCCTCTG
	FIP	GAGAGCAGAGCAAACCCCTCCACTGACTCCTCAGCAAAG
	BIP	GTTCCCACCCCGTTCGTCCTGAGCGCGTTGATGATC
	LF	TTCACCAGCTTGAGCCAG
	LB	GAGATCGAACGGCACCTC

### 3.2 Analytical sensitivity and specificity

**Figure 1:**
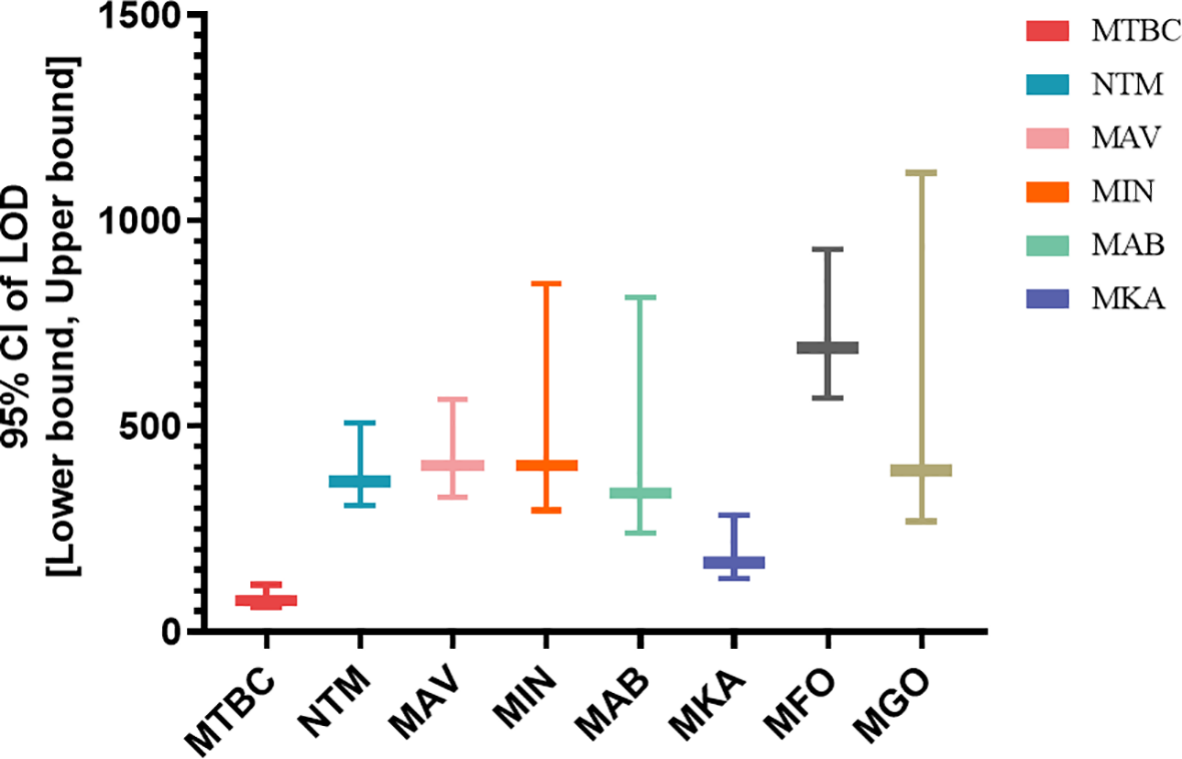
Limit of detection (LOD) of the multiplex LAMP assay for MTBC and major NTM species ^a^. ^a^LOD values with 95% confidence intervals (CIs) were determined by probit regression analysis using serial dilutions of quantified bacterial cultures spiked into negative sputum samples.

The analytical sensitivity of the assay was evaluated using serial dilutions of target organisms spiked into negative clinical sputum samples. Based on probit regression analysis, the assay demonstrated varying limits of detection (LODs) across different targets. The LOD for MTBC was 76.013 CFU/mL (95% CI: 60.329-113.924), indicating the highest sensitivity among all targets. For NTM, the LOD was 366.440 CFU/mL (95% CI: 305.954-506.989). Among specific NTM species, the calculated LOD values of the novel assay for *M. avium, M. intracellulare, M. kansasii*, *M. abscessus*, *M. gordonae* and *M. fortuitum* were found to be 403.564 CFU/mL (95% CI: 325.858-564.242 CFU/mL), 404.178 CFU/mL (95% CI: 295.311-846.424 CFU/mL), 166.602 CFU/mL (95% CI: 128.031-284.379 CFU/mL), 337.389 CFU/mL (95% CI: 240.078-811.646 CFU/mL), 391.61 CFU/mL (95% CI: 268.018-1116.418 CFU/mL), 690.629 CFU/mL (95% CI: 567.488-928.856 CFU/mL)(**Figure 1**). Overall, the assay exhibited high analytical sensitivity for MTBC and acceptable sensitivity for a wide range of clinically relevant NTM species. Furthermore, the specificity evaluation showed no cross-reactivity with non-mycobacterial respiratory pathogens, and all 28 tested mycobacterial strains were correctly identified, confirming the high specificity and inclusivity of the developed assay (**Supplementary Table S1**).

### 3.3 Performance on clinical samples

A total of 401 clinical samples, including 52 culture-confirmed isolates and 349 sputum specimens, were used to evaluate the performance of the assay. Among the 52 isolates, all NTM strains (52/52) were successfully detected. At the species level, the assay correctly identified all isolates of *M. intracellulare* (MIN, 18/18), *M. avium* (5/5), *M. kansasii* (10/10)*, M. fortuitum* (4/4), and *M. gordonae* (2/2). For *M. abscessus*, 12 out of 13 strains were detected. Sequencing analysis revealed that the undetected isolate was in fact *M. massiliense*, a subspecies within the *M. abscessus* complex (**Table 2**).

**Table 2:** Identification of NTM clinical isolates using the developed LAMP assay.

Species	No. of strains	No. correctly identified (%)	No. misidentified
NTM	52/52	52	0
*M. intracellulare*	18/18	18	0
*M. abscessus*	12/13	12	1(*M. massiliens*)
*M. kansasii*	10/10	10	0
*M. avium*	5/5	5	0
*M. fortuitum*	4/4	4	0
*M. gordonae*	2/2	2	0

In sputum samples, a total of 186 specimens were confirmed positive for MTBC by GeneXpert The assay detected 168 of these, corresponding to a sensitivity of 90.32% (95% CI: 85.14%-94.16%). Among the 163 reference-negative specimens, 159 were correctly identified, yielding a specificity of 97.55% (95% CI: 93.84%-99.33%). The overall agreement with the reference method was 93.70% (327/349; 95% CI: 90.61%-96.01%). *Kappa* analysis showed a strong agreement between the assay and GeneXpert, with a *Kappa* coefficient of 0.8740 (95% CI: 0.8233–0.9248), indicating good concordance (**Table 3**).

**Table 3:** Diagnostic performance of LAMP for the detection of MTBC from 349 sputum specimens compared with GeneXpert MTB/RIF.

The novel assay	Genexpert		Sensitivity % (95% CI)	Specificity % (95% CI)	OPA^a^ % (95% CI)	Cohen’s κ
	Positive	Negative				
Positive	168	4	90.32 (85.14-94.16)	97.55 (93.84-99.33)	93.70 (90.61-96.01)	0.8740
Negative	18	159				

a OPA, overall percent agreement.

**Figure 2:**
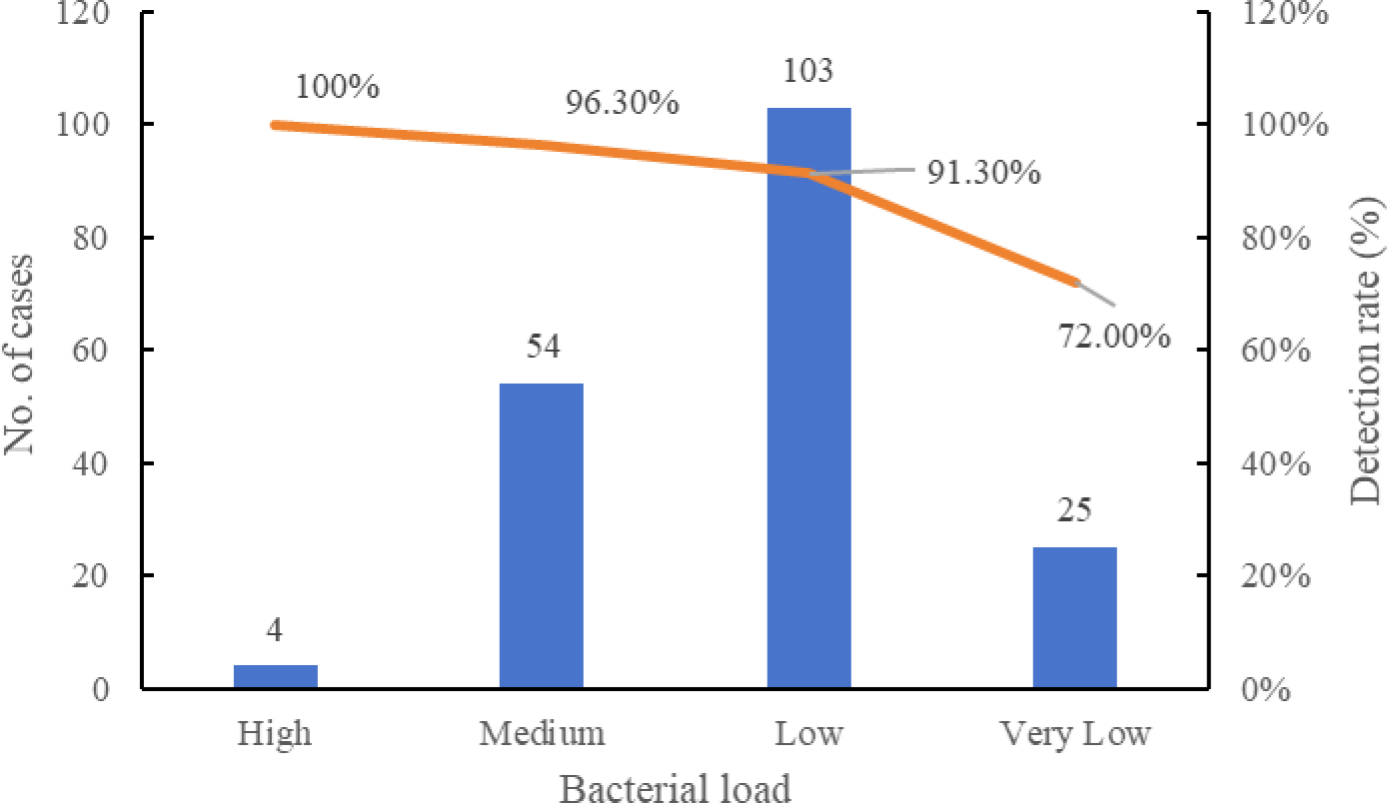
Detection rate of LAMP assay at different bacterial loads compared with Xpert ^a^. ^a^Sputum specimens positive by GeneXpert were stratified into High, Medium, Low, and Very Low categories. Detection rates of the LAMP assay were calculated for each category.

To further investigate the performance of the assay at different bacterial loads, we analyzed the detection rate of MTBC in clinical sputum samples stratified by Xpert semi-quantitative results. Among the 186 Xpert-positive specimens, the assay demonstrated consistently high detection rates, exceeding 90% across high to low bacterial load categories, whereas a marked decline was observed in the very low load group, with the rate decreasing to 72.0% (**Figure 2**). These results demonstrate that the assay maintains high sensitivity across a range of bacterial loads, particularly in moderate to high-load specimens. The reduced sensitivity observed in very low bacterial load samples is consistent with the inherent limitations of nucleic acid amplification tests in paucibacillary conditions and highlights the importance of specimen quality and bacterial concentration in achieving optimal diagnostic performance.

## 4 Discussion

Accurate and timely identification of *Mycobacterium* species is essential for appropriate clinical management, especially given the rising burden of NTM and their diverse antimicrobial susceptibilities. However, current commercial molecular kits often rely on conserved regions like 16S rRNA, hsp65 or ITS, which lack the discriminatory power to distinguish closely related species or subspecies. For instance, ITS-based assays cannot differentiate *M. massiliense* from *M. abscessus* (Nakanaga et al., 2014; Xiao et al., 2020), despite their distinct macrolide susceptibility profiles, leading to potential misdiagnosis and poor clinical outcomes, especially in the immunocompromised patients. To address these limitations, we developed a multiplex LAMP assay based on newly selected species-specific genetic targets identified through comparative genomic screening. Novel loci such as *XDL66245.1* (for *M. intracellulare*), *B1T47_13295* (for *M. kansasii*), and *dnaA* (for *M. abscessus*) demonstrated high inter-species specificity without homology to non-target strains. Compared with earlier LAMP-based assays, which often relied on pan-mycobacterial markers or genus-level resolution (Trangoni et al., 2015; Lapierre and Drancourt, 2018; Chen et al., 2021), our assay achieves finer species-level resolution and enhances clinical applicability.

Analytical performance confirmed that the assay could detect MTBC with high sensitivity (76.013 CFU/mL), surpassing that of many commercial NAATs such as GeneXpert MTB/RIF (113–131 CFU/mL) and loopamp MTBC detection kits (reported LOD ~100 CFU/mL) (Helb et al., 2010; Li et al., 2014). For NTM species, LODs ranged from 166.602 to 690.629 CFU/mL. Particularly, *M. kansasii* showed superior detection sensitivity, which is clinically relevant as this pathogen is frequently misdiagnosed as tuberculosis due to overlapping symptoms (Johnston et al., 2017). These results support the assay’s potential as both a primary and confirmatory diagnostic tool. In species-level testing of 52 cultured isolates, all *M. avium*, *M. intracellulare*, *M. fortuitum*, and *M. gordonae* strains were accurately identified. One undetected *M. abscessus* isolate was confirmed by sequencing to be *M. massiliense*, which lacks the targeted *erm(41)* region, further emphasizing the diagnostic gap in current assays and the necessity of precise subspecies-level differentiation.

The assay’s performance on 349 clinical sputum samples was similarly robust. Compared to GeneXpert, it showed a sensitivity of 90.32%, specificity of 97.55%, and overall agreement of 93.70% (*Kappa* = 0.8740). These values are consistent with previous LAMP-based evaluations and support the assay’s reliability in clinical settings (Yadav et al., 2022). Importantly, stratification by Xpert bacterial load showed that detection rates decreased with lower bacillary burden: from 100% (high load) to 72.0% (very low load), highlighting the expected challenge of diagnosing paucibacillary disease. This trend, however, is common among nucleic acid amplification techniques and does not significantly diminish the assay’s utility in routine diagnostics (Sabiiti et al., 2020). Unlike many commercial assays, our platform enables simultaneous detection of both MTBC and key NTM species, the isothermal nature of LAMP allows operation without a thermocycler, making it suitable for low-resource or decentralized settings. Its high amplification efficiency and inhibitor tolerance also enhance applicability to crude clinical specimens. In addition, the estimated cost of the multiplex LAMP assay is approximately $ 6~8/per test, which is lower than many commercial NAATs such as GeneXpert (∼$65). The workflow requires only basic pipetting skills and use of a portable fluorescence reader, allowing non-specialized personnel to be trained within 1~2 days. These features further support its feasibility for implementation in decentralized laboratories and resource-limited settings.

Nonetheless, the assay has certain limitations. The selection of NTM species was based on the distribution of clinical isolates at our institution between 2019 and 2021, including *M. gordonae*, which is frequently isolated from respiratory specimens and therefore relevant for demonstrating diagnostic discrimination from true pathogens. However, other clinically important or emerging NTM species (*M. simiae*, *M. xenopi*, *M. massiliense*) were not covered due to limited strain availability. During assay development, we also identified potential loci and designed primer sets for several additional species such as *M. chelonae*, *M. simiae*, and *M. marinum, etc*, but these could not be validated owing to the lack of representative clinical isolates. These targets will be incorporated in future studies to further expand the assay’s scope. In addition, given the substantial geographical variation in NTM prevalence, region-adapted panels that incorporate locally dominant species could further enhance the practical value of the assay and improve its relevance for both epidemiological surveillance and clinical management in diverse settings. The flexibility of the multiplex LAMP framework introduced in this study provides a feasible basis for tailoring diagnostic panels to region-specific needs, thereby extending its utility beyond single-institution validation. The false-negative case in *M. abscessus* also highlights to the need for target expansion at the subspecies level, particularly when resistance mechanisms are involved. Another limitation is the lack of whole-genome sequencing (WGS) validation to comprehensively confirm primer specificity and target stability, which will be addressed in future studies. Finally, although vitrification improved reagent stability under controlled laboratory conditions, data on long-term performance under variable field environments and crude sample inhibitor tolerance remain to be established.

Future refinements could further enhance the clinical utility of the assay. Incorporating resistance-associated loci such as *rpoB*, *katG*, and *rrl* would enable simultaneous species identification and resistance profiling. Most resistance-conferring mutations are SNP-based, which makes reliable discrimination between wild-type and mutant alleles difficult with dye-based LAMP alone. Such differentiation would require probe-based melting curve analysis, although probe design in LAMP is restricted to loop primer regions, thereby limiting flexibility. Finally, coupling the assay with microfluidic chip technology may provide a feasible strategy for field deployment. By automating sample pretreatment, nucleic acid extraction, and amplification within a single platform, such an approach could achieve a true “sample-in, answer-out” workflow, reducing manual handling, minimizing contamination risk, and facilitating use in decentralized or resource-limited settings.

In conclusion, this study introduces a novel, multiplex LAMP assay with good analytical and clinical performance. By incorporating new species-specific targets, the method improves the resolution and reliability of mycobacterial identification, addressing key limitations of current diagnostic platforms. Its speed, accuracy, and operational simplicity make it well-suited for routine use and point-of-care deployment, especially in high-burden or resource-limited regions. Given its robustness, simplicity, and field-deployability, the assay has strong potential to serve as a diagnostic tool in decentralized TB programs and to strengthen NTM surveillance efforts in low-resource settings.

## Data Availability

The original contributions presented in the study are included in the article/**Supplementary Material**. Further inquiries can be directed to the corresponding authors.
