# Supplementary material for: Rapid identification of major Mycobacterium species by loop-mediated isothermal amplification assay using novel species-specific genomic targets
Source: Front Cell Infect Microbiol. 2025 Sep 17;15:1653602. doi: 10.3389/fcimb.2025.1653602 (PMC12484021; doi:10.3389/fcimb.2025.1653602)
Supplement: Supplementary file 1 [file Table1.docx]

Table S1 Bacteria used in this study.

| Species | Number | Source | Results |
| --- | --- | --- | --- |
| *Mycobacterium tuberculosis* | 12 | NIFDC | + (12/12) |
| *Mycobacterium avium* | 2 | NIFDC and BNCC | +（2/2） |
| *Mycobacterium intracellulare* | 1 | BNCC | +（1/1） |
| *Mycobacterium abscessus* | 2 | NIFDC and BNCC | +（2/2） |
| *Mycobacterium fortuitum* | 2 | NIFDC and BNCC | +（2/2） |
| *Mycobacterium gordonae* | 2 | NIFDC and BNCC | +（2/2） |
| *Mycobacterium kansasii* | 2 | NIFDC and BNCC | +（2/2） |
| *Mycobacterium terrae* | 1 | NIFDC | -(16S +) |
| *Mycobacterium chelonae* | 1 | BNCC | -(16S +) |
| *Mycobacterium asiaticum* | 1 | NIFDC | -(16S +) |
| *Mycobacterium szulgai* | 1 | BNCC | -(16S +) |
| *Mycobacterium scrofulaceum* | 1 | NIFDC | -(16S +)- |
| *Pseudomonas aeruginosa* | 3 | BNCC and clinical isolates | - |
| *Staphylococcus aureus* | 2 | BNCC and clinical isolates | - |
| *Nocardia brasiliensis* | 1 | NIFDC | - |
| *Legionella pneumophila* | 1 | BNCC | - |
| *Klebsiella pneumoniae* | 1 | BNCC | - |
| *Streptococcus pneumoniae* | 1 | BNCC | - |
| *Haemophilus influenzae* | 1 | BNCC | - |
| *Bordetella pertussis* | 1 | NIFDC |  |

NIFDC: National Institutes for Food and Drug Control BNCC: BeNa Culture Collection. +, positive result. -, negative result.

Table S2 Species-specific nucleic acid sequence for each NTM strain and MTBC were selected through comparative genomic analysis and published studies

| Species | Target | Sequence |
| --- | --- | --- |
| *M. tuberculosis complex* | *IS6110* | CGGTCGGAGCGGTCGGAAGCTCCTATGACAATGCACTAGCCGAGACGATCAACGGCCTATACAAGACCGAGCTGATCAAACCCGGCAAGCCCTGGCGGTCCATCGAGGATGTCGAGTTGGCCACCGCGCGCTGGGTCGACTGGTTCAACCATCGCCGCCTCTACCAGTACTGCGGCGACGTCCCGCCGGTCGAACTCGAGGCTGCCTACTACGCTCAACGCCAGAGACCA |
| *Mycobacterium spp.* | *16S rRNA* | CCGCGGTAATACGTAGGGTCCGAGCGTTGTCCGGAATTACTGGGCGTAAAGAGCTCGTAGGTGGTTTGTCGCGTTGTTCGTGAAAACTCACAGCTTAACTGTGGGCGTGCGGGCGATACGGGCAGACTAGAGTACTGCAGGGGAGACTGGAATTCCTGGTGTAGCGGTGGAATGCGCAGATATCAGGAGGAACACCGGT |
| *M. intracellulare* | *MINTM006_47990* | GCCGCTATCTTCCGTGGTACGAGAGCAACCCATACATGGCGACCTTGCTGTTCCCCCTAATCGCGATCGCCATGATTGGCGCAGGATGTGCACCCGGTAATCCGGCGTGGCTGTGGTACGGCGGGGCCATTCTGCTGGCCATCTCTGCCTTATTGGTGGGAGTCTCCCTCTGGATCTGGCGCCGATCCCTTCTCCGTATCACCCC |
| *M. avium* | *gyrB* | GCCTGACCATCAACCTCACCGACGAGCGGGTGACCAACGAAGAGGTCGTCGACGAGGTGGTCAGCGACACCGCCGACGCACCCAAGTCGGCGCAGGAGAAGGCGGCGGAATCGGCTGCGCCGCATAAGGTCAAGCACCGCACCTTCCACTACCCCGGCGGCCTGGTCGACTTCGTCAAACACATCAATCGCACCAAAAACCCCATCCACCA |
| *M. abscessus* | *erm(41)* | TGCCAGGGTGCTAGCCGTCGAGCTGCATCCGGGGCGGGCTCGACACCTTCGTTCACGGTTTGCCGAGGAAGATGTCCGGATAGCGGAAGCGGACCTGCTCGCCTTCCGGTGGCCGCGACGGCCATTTCGGGTGGTGGCGAGCCCGCCCTACCAAGTCACCAGCGCACTGATACGGAGTCTCTTGACGCCGGAATCCCGGCTGCTGGCTGCCGACCTGGTGCTGCAGCGCGGGGCTGTGCACAAACATGCGAAGCGAGCACCTGTTCGCCA |
| *M. kansasii* | *hypothetical protein B1T47_13295* | CCCGGTCCACTATTACGACATTCCGCTGCGTGGGCCGATATTCAGAATCGACCGCATGGTCCGCCGCAACCTGCATCGCACGGCAACCGATCAACGGTTCCTCTTGGGCGCCAACATGGCAATCCGGACCTCGGCGTGGCAGGCGGTACGTCATCTCACGCAGCTGGATCTGGAAGACCGACTCCACGAAGACATCGATCTTGCACTGACACTG |
| *M. fortuitum* | *dnaA* | GATCGTGATTCCAACAGTGATCCGGCGCTCCCACCACTGACTCCTCAGCAAAGAGCCTGGCTCAAGCTGGTGAAACCCCTCGTCATCGCCGAGGGGTTTGCTCTGCTCTCCGTTCCCACCCCGTTCGTCCAGAACGAGATCGAACGGCACCTCCGCGAACCGATCATCAACGCGCTCAGCCGCAAGCTTGGTCAACGCGTCGAGCTGGGCGTACGCATCGCCACCCCCCCAGAGGAATCCGAGGACT |
| *M. gordonae* | ITS | ATCCACCATGCGCCCTTAGACACTTACAAACACTACAAAAACCAAAGAATAAAATTGCACAAAAAGAACACGTTGCCGCGAACGACAACGCATACATTTTGATGCTCGCAACCACTATCCAATTCTCAAACACCACACCCCACCACCAAGATGGAGGGACAGCACCCGAGGGTGTTGCCTCAGGACCCAATAGTGTGTCTGGCTTTGCCTGTTGTCGTGCACCCGGTCTTCGTCCACTACAGACGATGACCCCTCACGGCTCGCACCCCACCAATTGGAGTGCTCTTCGTGGTGCTCCTTAGAAAGGAGGTGAT^a^ |

^a^ The red-marked sequences indicate regions where at least one segment of the FIP or BIP must be located in the LAMP primer sets.
